# Association between triglyceride glucose index-related indices with gallstone disease among US adults

**DOI:** 10.1186/s12944-024-02194-x

**Published:** 2024-06-27

**Authors:** Chang Fu, Xiaocong Li, Yongxin Wang, Junhong Chen, Yibo Yang, Kai Liu

**Affiliations:** 1 Department of Hepatobiliary and Pancreatic Surgery, General Surgery Center, The First Hospital of Jilin University, Changchun, Jilin 130021 China; 2 Department of Pharmacy, China-Japan Friendship Hospital, Beijing, 100020 China

**Keywords:** Gallstone disease, Triglyceride glucose-body mass index (TyG-BMI), Triglyceride glucose-waist to height ratio (TyG-WHtR), Triglyceride glucose-waist circumference (TyG-WC), National Health and Nutrition Examination Survey (NHANES)

## Abstract

**Background:**

Triglyceride glucose (TyG) index combined with obesity-related indicators [triglyceride glucose-body mass index (TyG-BMI), triglyceride glucose-waist to height ratio (TyG-WHtR), triglyceride glucose-waist circumference (TyG-WC)], represents emerging methodologies for assessing insulin resistance. The objective of this investigation was to explore the correlation between TyG-related indices and gallstone disease.

**Methods:**

The study included 3740 adults from the 2017–2020 period of the National Health and Nutrition Examination Survey. TyG-BMI, TyG-WC, and TyG-WHtR were integrated as both continuous and categorical variables within the multivariate logistic model, respectively to evaluate the connection between various TyG-related indices and gallstone disease. Additionally, restriction cubic splines and subgroup analysis were employed to deepen our understanding of this relationship.

**Results:**

When analyzed as continuous variables, positive correlations were observed between TyG-BMI, TyG-WC, TyG-WHtR and gallstone disease. The OR(95%CI) were 1.063(1.045,1.082) for TyG-BMI (per 10-unit), 1.026(1.018,1.034) for TyG-WC (per 10-unit) and 1.483(1.314,1.676) for TyG-WHtR (per 1-unit), respectively. When categorized into quartiles, these three TyG-related indices still show statistically significant associations with gallstone disease. Descending in order, the diagnostic capability for gallstone disease is demonstrated as follows: TyG-WHtR (AUC = 0.667), TyG-BMI (AUC = 0.647), and TyG-WC (AUC = 0.640).

**Conclusion:**

There were significantly positive associations between TyG-related indices, including TyG-BMI, TyG-WC, and TyG-WHtR, and gallstone disease. Of these indices, TyG-WHtR demonstrated the most favorable performance in identifying the risk of gallstone disease.

## Introduction

Gallstone disease is the most common disease in the hepatobiliary system, imposing a significant economic and medical burden on society [1, 2]. Frequently accompanied by cholecystitis, it can escalate to severe acute conditions such as bile duct stones and pancreatitis, thereby posing substantial health risks to patients [3]. Moreover, gallstone disease represents a significant risk factor for the development of gallbladder cancer, which has a very poor prognosis [4–6]. Risk factors for gallstone disease encompass advanced age, female gender, genetic predisposition, metabolic syndrome, insulin resistance (IR), and lifestyle factors [7]. Therefore, the exploration of effective clinical indicators for the early detection and intervention of gallstone disease risk factors holds paramount importance in mitigating this global public health burden.

IR is a prominent characteristic of metabolic syndrome, marked by reduced physiological response to insulin within the body [8]. While the hyperinsulinemic-euglycemic clamp (HEC) test is considered the gold standard for measuring IR, its complexity and invasiveness constrain its practical application in clinical settings [9]. An alternative method for assessing IR is homeostasis model assessment of insulin resistance (HOMA-IR), but it has the disadvantage of requiring the measurement of fasting insulin level [10]. Triglyceride glucose (TyG) index emerges as a novel parameter integrating triglyceride and glucose to assess IR [11]. In comparison to the conventional methods mentioned earlier, the TyG index offers increased feasibility and cost-effectiveness in clinical applications. Research have found that TyG-related indices, such as triglyceride glucose-body mass index (TyG-BMI), triglyceride glucose-waist-to-height ratio (TyG-WHtR), and triglyceride glucose-waist circumference (TyG-WC), which amalgamate obesity-related metrics including body mass index (BMI), waist-to-height ratio (WHtR), and waist circumference (WC), offer enhanced assessment of IR [12–14]. The utility of these indices in assessing the risk of metabolic syndrome, metabolic dysfunction associated fatty liver (MAFLD) and cardiovascular disease (CVD) has been validated [15–18]. However, investigations into the association between TyG-related indices and gallstone disease remain lacking.

Our study explored the correlation between TyG-related indices and gallstone disease through the National Health and Nutrition Examination Survey (NHANES) database, aiming to provide more practical clinical indicators for assessing the risk of gallstone disease.

## Materials and methods

### Study design

**Fig. 1:**
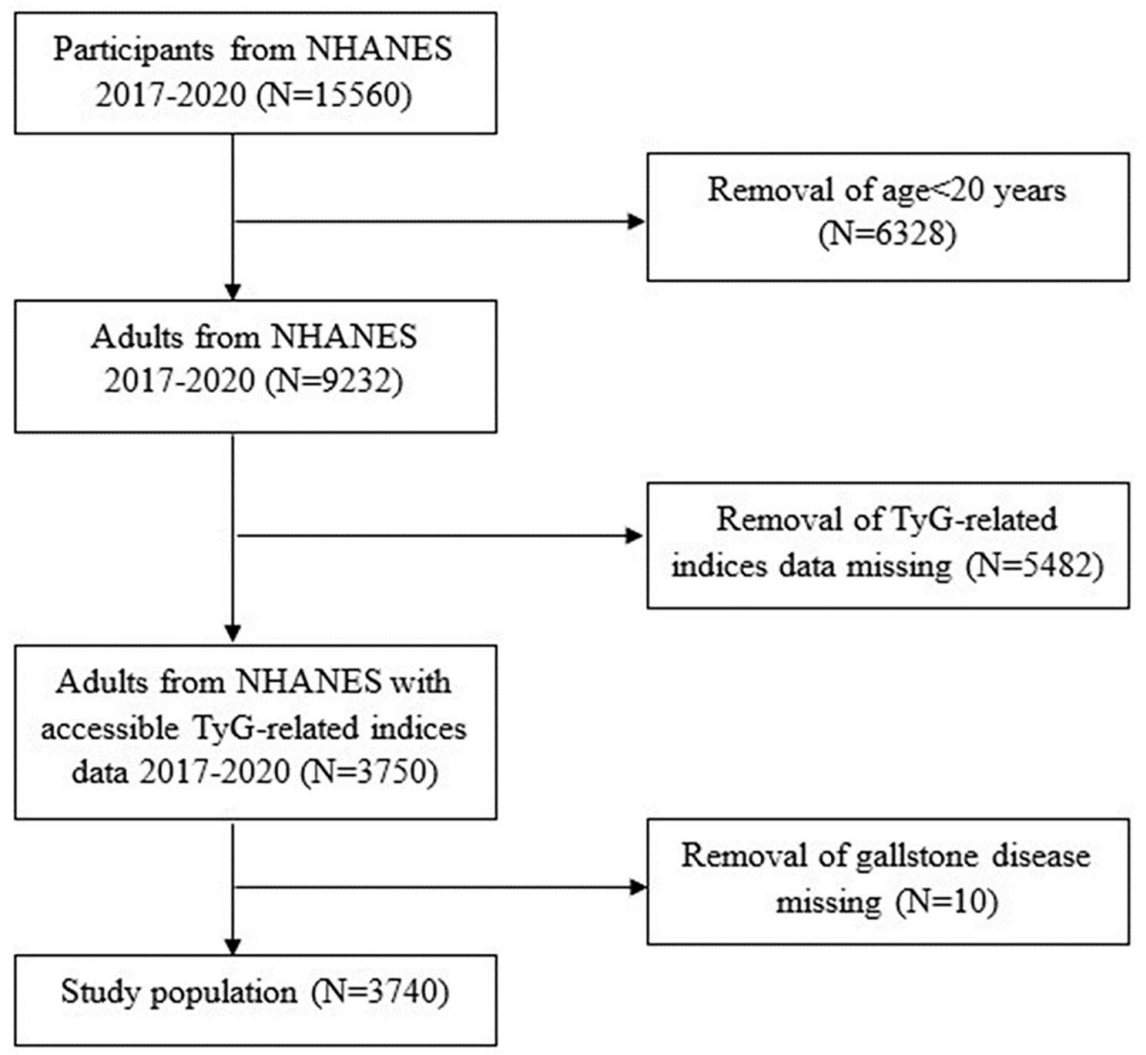
Flow chart for screening the study population

The NHANES is a research initiative undertaken by the National Center for Health Statistics (NCHS) aimed at evaluating the health and nutritional well-being of both adults and children across the United States. The NCHS Ethics Review Board granted ethical approval for the survey, and participants furnished written informed consent before participation. In our investigation, data extraction was conducted from the NHANES database spanning the years 2017 to March 2020, as the relevant questionnaires for gallstone disease were available only during this period. The exclusion criteria were delineated as follows: [1] age < 20 years old; [2] missing data on triglycerides (TG); [3] missing data on gallstone disease; [4] missing data on BMI and WC. Following screening based on these criteria, 3740 individuals were ultimately selected from the original pool of 15,560 participants (Fig. 1).

### Study variables

The TyG index and TyG-related indices were calculated as follows:

$$TyG={ln}\left[\frac{TG(mg/dl)\times glucose(mg/dl)}{2}\right]$$

$$TyG-BMI=TyG\times BMI$$

$$TyG-WHtR=TyG\times WHtR, WHtR=\frac{WC}{height}$$

$$TyG-WC=TyG\times WC$$

Measurements of body weight, WC and height were conducted by trained health technicians in the Mobile Examination Center (MEC). Fasting blood samples were collected on-site, stored under appropriate conditions and transported to the laboratory for analysis. The diagnosis of gallstone disease was based on the participant’s response to the questionnaire “Has a doctor or other health professional ever told you that you had gallstones?”

The covariates considered in our analysis encompassed age, gender, race, education level, marital status, poverty income ratio (PIR), diabetes, hypertension, smoking and drinking status. We operationalized education level by defining high school completion as the cutoff point and categorized it into three groups: less than high school, high school, and more than high school. PIR is calculated by dividing household income by the poverty line in the survey year and is regarded as the main indicator of socioeconomic status [19]. The prevalence of diabetes and hypertension were determined by assessing participants’ responses to the questionnaires (DIQ020 and BPQ020). Participants’ smoking and drinking statuses were ascertained based on the outcomes derived from the questionnaires (SMQ020 and ALQ101) they completed. Information regarding the methods employed for variable collection can be referenced in the NHANES Survey Methods and Analysis Guide.

### Statistical analysis

Normally distributed continuous variables were described by mean ± standard deviation (mean ± sd), while non-normally distributed data were represented by the median (interquartile range [IQR]). Categorical variables were depicted in numbers (n) and percentages (%). Continuous variables were analyzed using both ANOVA and Kruskal-Wallis H tests, while categorical variables were assessed using chi-square tests to compare the characteristics of study population with and without gallstone disease. Participants in the study were categorized into quartiles based on their TyG-BMI, TyG-WC, and TyG-WHtR values, denoted as Q1, Q2, Q3, and Q4. In three models, multivariate logistic regression was utilized to evaluate the relationship between various TyG-related indices and gallstone disease, with odds ratios (OR) and 95% confidence intervals (CI) employed to indicate the magnitude of these associations. Model 1 did not include any covariates, whereas Model 2 incorporated gender, age, and race as covariates. The fully adjusted model (Model 3) further adjusted for age, gender, race, education level, marital status, PIR, diabetes, hypertension, smoke and drinking status. Initially, TyG-related indices were introduced as continuous variables in logistic models, subsequently categorized into quartiles. Restricted cubic splines (RCS) were utilized to investigate potential nonlinear dose-response relationships between TyG-related indices and gallstone disease. Subgroup analysis was conducted to investigate potential differences across different subgroups. The receiver operating characteristic (ROC) curve was utilized to compare the predictive performance of the three indexes, and the cutoff values and corresponding sensitivity and specificity were further explored. Statistical analyses were performed using SAS version 9.4, with statistical significance set at *P* < 0.05.

## Results

### Characteristics of the participants

Among the 3740 participants included in this analysis, 397 individuals had gallstone disease, representing a prevalence of 10.6%. The mean ± SD of TyG and TyG-related indices (TyG-BMI, TyG-WC, and TyG-WHtR) were 8.5 ± 0.7, 255.4 ± 70.5, 862.1 ± 181.9 and 5.2 ± 1.1, respectively. Table 1 presents the characteristics of the participants both with and without gallstone disease. Generally, people with gallstone disease tends to be older, more commonly females, and exhibited a higher prevalence of diabetes and hypertension. Drinkers more prevalent among those without gallstone disease. Additionally, individuals diagnosed with gallstone disease exhibited markedly elevated levels of TyG and TyG-related indices in comparison to those without the condition (*P* < 0.0001).

**Table 1:** Comparison of characteristics of the study population with and without gallstone disease

Characteristics	Total (*N* = 3740)	Without gallstone disease (*N* = 3343)	With gallstone disease (*N* = 397)	*P* Value
**Age (year), mean ± SD**	50.7 ± 17.3	49.9 ± 17.3	57.4 ± 15.4	< 0.0001
**Gender, n (%)**				< 0.0001
Male	1815 (48.5)	1703 (50.9)	112 (28.2)	
Female	1925 (51.5)	1640 (49.1)	285 (71.8)	
**Race, n (%)**				0.0002
Mexican American	481 (12.9)	423 (12.7)	58 (14.6)	
Other Hispanic	380 (10.2)	328 (9.8)	52 (13.1)	
Non-Hispanic White	1268 (33.9)	1111 (33.2)	157 (39.5)	
Non-Hispanic Black	943 (25.2)	875 (26.2)	68 (17.1)	
Other Race	668 (17.9)	606 (18.1)	62 (15.6)	
**Education level, n (%)**				00.6732
Less than high school	707 (18.9)	628 (18.8)	79 (19.9)	
High school	891 (23.8)	792 (23.7)	99 (24.9)	
More than high school	2140 (57.2)	1921 (57.5)	219 (55.2)	
**Marital status, n (%)**				0.9735
Cohabitation	2218 (59.4)	1982 (59.4)	236 (59.4)	
Solitude	1518 (40.6)	1357 (40.6)	161 (40.6)	
**Diabetes, n (%)**				< 0.0001
Yes	596 (15.9)	490 (14.7)	106 (26.7)	
No	3142 (84.1)	2851 (85.3)	291 (73.3)	
**Hypertension, n (%)**				< 0.0001
Yes	1436 (38.4)	1220 (36.5)	216 (54.4)	
No	2299 (61.6)	2118 (63.5)	181 (45.6)	
**Smoking status, n (%)**				0.3292
Yes	1608 (43.0)	1429 (42.7)	179 (45.3)	
No	2130 (57.0)	1914 (57.3)	216 (54.7)	
**Drinking status, n (%)**				< 0.0001
Yes	1646 (50.5)	1511 (51.8)	135 (39.1)	
No	1615 (49.5)	1405 (48.2)	210 (60.9)	
**PIR, mean ± SD**	2.6 ± 1.6	2.6 ± 1.6	2.5 ± 1.5	0.3505
**BMI (kg/m** ^**2**^ **), mean ± SD**	29.8 ± 7.3	29.4 ± 7.0	33.2 ± 8.5	< 0.0001
**Triglyceride (mg/dL), mean ± SD**	110.0 ± 95.3	108.6 ± 95.2	121.7 ± 95.0	0.0098
**Fasting blood glucose (mg/dL), mean ± SD**	113.4 ± 37.9	112.6 ± 37.4	120.3 ± 41.2	0.0001
**Stand height (cm), mean ± SD**	166.9 ± 10.0	167.3 ± 10.0	163.8 ± 9.0	< 0.0001
**Waist circumference (cm), mean ± SD**	100.8 ± 17.1	99.9 ± 16.9	108.1 ± 17.3	< 0.0001
**TyG, mean ± SD**	8.5 ± 0.7	8.5 ± 0.7	8.7 ± 0.7	< 0.0001
**TyG-BMI, mean ± SD**	255.4 ± 70.5	251.2 ± 68.0	290.0 ± 81.4	< 0.0001
**TyG-WC, mean ± SD**	862.1 ± 181.9	852.4 ± 179.2	943.6 ± 184.3	< 0.0001
**TyG-WHtR, mean ± SD**	5.2 ± 1.1	5.1 ± 1.1	5.8 ± 1.1	< 0.0001

*PIR: the ratio of income to poverty; BMI, body mass index; TyG: triglyceride glucose index; TyG-BMI: triglyceride glucose-body mass index; TyG-WC: triglyceride glucose-waist circumference; TyG-WHtR: triglyceride glucose-waist to height ratio

Participants in the study were divided into quartiles according to TyG-BMI, TyG-WC, and TyG-WHtR, and the characteristics of these different subgroups were compared and presented in Table 2. Regardless of which of the three TyG-related indices was used for grouping, it was found that the subgroups with higher indices exhibited a greater proportion of individuals with hypertension and diabetes, and a relatively low proportion of people drinking alcohol. Trends in BMI, triglyceride, fasting blood glucose, WC, and TyG were also consistent with changes in the TyG-related indices.

**Table 2:** Differences among groups were compared according to TyG-related indices

	TyG-BMI					TyG-WC					TyG-WHtR				
Characteristics	Q1 (< 205.8) (*N* = 935)	Q2 (205.8-244.9) (*N* = 935)	Q3 (244.9-293.1) (*N* = 935)	Q4 (> 293.1) (*N* = 935)	*P* Value	Q1 (< 731.9) (*N* = 935)	Q2 (731.9-849.3) (*N* = 935)	Q3 (849.3-979.2) (*N* = 935)	Q4 (> 979.2) (*N* = 935)	*P* Value	Q1 (< 4.39) (*N* = 935)	Q2 (4.39–5.11) (*N* = 935)	Q3 (5.11–5.86) (*N* = 935)	Q4 (> 5.86) (*N* = 935)	*P* Value
**Age (year), mean ± SD**	45.8 ± 18.4	53.8 ± 17.0	52.6 ± 16.7	50.6 ± 15.9	< 0.0001	42.8 ± 17.4	52.9 ± 16.9	53.7 ± 16.6	53.4 ± 15.8	< 0.0001	42.3 ± 16.9	52.3 ± 16.8	53.9 ± 16.8	54.3 ± 15.8	< 0.0001
**Gender, n (%)**					< 0.0001					< 0.0001					< 0.0001
Male	426 (45.6)	518 (55.4)	472 (50.5)	399 (42.7)		368 (39.4)	456 (48.8)	497 (53.2)	494 (52.8)		465 (49.7)	506 (54.1)	469 (50.2)	375 (40.1)	
Female	509 (54.4)	417 (44.6)	463 (49.5)	536 (57.3)		567 (60.6)	479 (51.2)	438 (46.8)	441 (47.2)		470 (50.3)	429 (45.9)	466 (49.8)	560 (59.9)	
**Race, n (%)**					< 0.0001					< 0.0001					< 0.0001
Mexican American	71 (7.6)	117 (12.5)	145 (15.5)	148 (15.8)		74 (7.9)	139 (14.9)	125 (13.4)	143 (15.3)		73 (7.8)	112 (12.0)	141 (15.1)	155 (16.6)	
Other Hispanic	66 (7.1)	99 (10.6)	112 (12.0)	103 (11.0)		68 (7.3)	113 (12.1)	106 (11.3)	93 (9.9)		64 (6.8)	100 (10.7)	104 (11.1)	112 (12.0)	
Non-Hispanic White	307 (32.8)	319 (34.1)	323 (34.5)	319 (34.1)		281 (30.1)	277 (29.6)	337 (36.0)	373 (39.9)		297 (31.8)	296 (31.7)	325 (34.8)	350 (37.4)	
Non-Hispanic Black	259 (27.7)	200 (21.4)	218 (23.3)	266 (28.4)		266 (28.4)	228 (24.4)	219 (23.4)	230 (24.6)		292 (31.2)	225 (24.1)	213 (22.8)	213 (22.8)	
Other Race	232 (24.8)	200 (21.4)	137 (14.7)	99 (10.6)		246 (26.3)	178 (19.0)	148 (15.8)	96 (10.3)		209 (22.4)	202 (21.6)	152 (16.3)	105 (11.2)	
**Education level, n (%)**					0.0034					< 0.0001					< 0.0001
Less than high school	135 (14.4)	187 (20.0)	200 (21.4)	185 (19.8)		116 (12.4)	195 (20.9)	209 (22.4)	187 (20.0)		108 (11.6)	175 (18.8)	203 (21.7)	221 (23.6)	
High school	229 (24.5)	215 (23.0)	210 (22.5)	237 (25.3)		240 (25.7)	193 (20.7)	210 (22.5)	248 (26.5)		224 (24.0)	206 (22.1)	228 (24.4)	233 (24.9)	
More than high school	571 (61.1)	531 (56.9)	525 (56.1)	513 (54.9)		579 (61.9)	545 (58.4)	516 (55.2)	500 (53.5)		603 (64.5)	552 (59.2)	504 (53.9)	481 (51.4)	
**Marital status, n (%)**					< 0.0001					0.0092					< 0.0001
Cohabitation	495 (53.1)	607 (65.0)	579 (61.9)	537 (57.5)		512 (54.9)	578 (61.9)	572 (61.2)	556 (59.5)		506 (54.2)	591 (63.3)	584 (62.5)	537 (57.5)	
Solitude	438 (46.9)	327 (35.0)	356 (38.1)	397 (42.5)		421 (45.1)	356 (38.1)	363 (38.8)	378 (40.5)		427 (45.8)	343 (36.7)	351 (37.5)	397 (42.5)	
**PIR, mean ± SD**	2.7 ± 1.6	2.8 ± 1.6	2.6 ± 1.6	2.4 ± 1.5	< 0.0001	2.8 ± 1.6	2.7 ± 1.6	2.6 ± 1.6	2.4 ± 1.5	0.0002	2.8 ± 1.7	2.8 ± 1.6	2.6 ± 1.6	2.3 ± 1.5	< 0.0001
**Diabetes, n (%)**					< 0.0001					< 0.0001					< 0.0001
Yes	51 (5.5)	106 (11.3)	162 (17.3)	277 (29.6)		32 (3.4)	96 (10.3)	147 (15.7)	321 (34.3)		30 (3.2)	88 (9.4)	155 (16.6)	323 (34.5)	
No	884 (94.5)	828 (88.7)	772 (82.7)	658 (70.4)		902 (96.6)	839 (89.7)	787 (84.3)	614 (65.7)		904 (96.8)	847 (90.6)	779 (83.4)	612 (65.5)	
**Hypertension, n (%)**					< 0.0001					< 0.0001					< 0.0001
Yes	193 (20.6)	337 (36.2)	408 (43.7)	498 (53.3)		169 (18.1)	339 (36.4)	408 (43.7)	520 (55.6)		160 (17.1)	340 (36.4)	404 (43.3)	532 (57.0)	
No	742 (79.4)	595 (63.8)	525 (56.3)	437 (46.7)		766 (81.9)	592 (63.6)	526 (56.3)	415 (44.4)		775 (82.9)	593 (63.6)	529 (56.7)	402 (43.0)	
**Smoking status, n (%)**					0.0558					< 0.0001					< 0.0001
Yes	381 (40.8)	391 (41.8)	400 (42.8)	436 (46.7)		342 (36.6)	374 (40.0)	414 (44.3)	478 (51.1)		378 (40.5)	372 (39.8)	421 (45.0)	437 (46.8)	
No	553 (59.2)	544 (58.2)	535 (57.2)	498 (53.3)		592 (63.4)	561 (60.0)	520 (55.7)	457 (48.9)		556 (59.5)	563 (60.2)	514 (55.0)	497 (53.2)	
**Drinking status, n (%)**					< 0.0001					< 0.0001					< 0.0001
Yes	455 (56.6)	407 (50.7)	413 (50.8)	371 (44.1)		448 (56.4)	420 (52.0)	409 (49.7)	369 (44.2)		485 (59.5)	430 (52.6)	416 (51.2)	315 (38.7)	
No	349 (43.4)	395 (49.3)	400 (49.2)	471 (55.9)		347 (43.6)	388 (48.0)	414 (50.3)	466 (55.8)		330 (40.5)	388 (47.4)	397 (48.8)	500 (61.3)	
**BMI (kg/m** ^**2**^ **), mean ± SD**	22.3 ± 2.4	26.9 ± 2.0	30.9 ± 2.5	39.3 ± 6.4	< 0.0001	23.0 ± 3.2	27.4 ± 3.5	30.9 ± 4.2	38.0 ± 7.2	< 0.0001	23.0 ± 3.2	27.4 ± 3.4	30.9 ± 4.2	38.0 ± 7.3	< 0.0001
**Triglyceride (mg/dL), mean ± SD**	65.2 ± 32.2	98.1 ± 51.9	121.5 ± 71.9	155.2 ± 152.0	< 0.0001	62.4 ± 29.6	91.8 ± 45.1	120.4 ± 66.1	165.5 ± 152.5	< 0.0001	61.2 ± 29.2	92.7 ± 45.5	119.3 ± 63.9	167.0 ± 152.6	< 0.0001
**Fasting blood glucose (mg/dL), mean ± SD**	99.1 ± 14.5	108.1 ± 27.1	114.7 ± 36.2	131.7 ± 54.0	< 0.0001	98.0 ± 12.6	106.0 ± 22.6	112.1 ± 29.1	137.6 ± 57.8	< 0.0001	98.1 ± 13.6	106.0 ± 22.0	112.5 ± 28.4	137.1 ± 58.4	< 0.0001
**Stand height (cm), mean ± SD**	167.0 ± 9.9	167.1 ± 10.0	166.8 ± 10.3	166.6 ± 9.6	0.6656	165.6 ± 9.7	166.0 ± 10.2	167.4 ± 9.7	168.5 ± 9.9	< 0.0001	168.8 ± 9.8	167.4 ± 9.8	166.4 ± 10.2	164.9 ± 9.7	< 0.0001
**Waist (cm), mean ± SD**	81.9 ± 7.9	95.2 ± 7.1	104.8 ± 8.6	121.2 ± 13.0	< 0.0001	81.1 ± 7.1	95.1 ± 5.9	105.2 ± 7.2	121.7 ± 12.6	< 0.0001	82.1 ± 8.3	95.4 ± 7.6	105.2 ± 9.0	120.4 ± 13.7	< 0.0001
**TyG, mean ± SD**	8.0 ± 0.5	8.4 ± 0.5	8.7 ± 0.6	9.0 ± 0.7	< 0.0001	7.9 ± 0.5	8.4 ± 0.5	8.7 ± 0.5	9.1 ± 0.7	< 0.0001	7.9 ± 0.4	8.4 ± 0.5	8.7 ± 0.5	9.1 ± 0.7	< 0.0001
**TyG-BMI, mean ± SD**	177.7 ± 19.7	225.8 ± 11.1	266.9 ± 14.0	351.1 ± 54.6	< 0.0001	181.6 ± 25.4	228.7 ± 23.9	266.8 ± 28.8	344.3 ± 60.5	< 0.0001	181.5 ± 24.9	228.9 ± 23.3	267.0 ± 29.1	344.1 ± 60.9	< 0.0001
**TyG-WC, mean ± SD**	652.6 ± 76.1	802.1 ± 64.5	907.6 ± 77.3	1086.2 ± 129.1	< 0.0001	641.9 ± 62.2	793.4 ± 33.5	909.3 ± 36.8	1103.9 ± 109.9	< 0.0001	648.6 ± 71.4	798.0 ± 55.8	909.2 ± 65.0	1092.7 ± 122.0	< 0.0001
**TyG-WHtR, mean ± SD**	3.9 ± 0.5	4.8 ± 0.4	5.5 ± 0.4	6.5 ± 0.8	< 0.0001	3.9 ± 0.4	4.8 ± 0.4	5.4 ± 0.4	6.6 ± 0.7	< 0.0001	3.8 ± 0.4	4.8 ± 0.2	5.5 ± 0.2	6.6 ± 0.7	< 0.0001

### Associations between TyG-related indices and gallstone disease

In Table 3, the associations linking TyG-BMI, TyG-WC, TyG-WHtR, and gallstone disease were presented as OR with corresponding 95%CI. Upon analyzing the TyG-related indices as continuous variables, a positive correlation was evident between TyG-BMI, TyG-WC, TyG-WHtR and gallstone disease in all three models (*P* < 0.05). In the fully adjusted model, a 10-unit rise in TyG-BMI was associated with a 6.3% increase in the risk of developing gallstone disease [OR (95%CI): 1.063(1.045,1.082)], Similarly, for each 10-unit rise in TyG-WC, there is a 2.6% increase in the risk of gallstone disease [OR (95%CI): 1.026(1.018,1.034)]. In Model 3, the risk of gallstone disease escalated by 48.3% [OR (95%CI): 1.483(1.314,1.676)] with every 1-unit increment in TyG-WHtR. When grouped into quartiles, these three TyG-related indices still show statistically significant associations with gallstone disease. Compared to those in the Q1 group, those with higher TyG-related indices had an elevated risk of gallstone disease and showed an increasing trend, with TyG-WC exerting the greatest effect.

**Table 3:** Association between TyG-BMI, TyG-WC, TyG-WHtR and gallstone disease

Exposure	Model 1 OR(95%CI)	Model 2 OR(95%CI)	Model 3 OR(95%CI)
TyG-BMI (per 10 units)	1.069(1.055,1.084)	1.071(1.056,1.086)	1.063(1.045,1.082)
TyG-BMI (quartile)			
Q1	1.00(Reference)	1.00(Reference)	1.00(Reference)
Q2	1.825(1.251,2.663)	1.609(1.091,2.372)	1.755(1.103,2.794)
Q3	2.636(1.840,3.777)	2.351(1.622,3.406)	2.515(1.610,3.929)
Q4	4.175(2.960,5.888)	3.926(2.752,5.600)	3.591(2.314,5.572)
TyG-WC (per 10 units)	1.026(1.021,1.032)	1.028(1.022,1.034)	1.026(1.018,1.034)
TyG-WC (quartile)			
Q1	1.00(Reference)	1.00(Reference)	1.00(Reference)
Q2	2.383(1.623,3.499)	2.116(1.423,3.147)	2.709(1.665,4.407)
Q3	3.045(2.096,4.423)	2.843(1.929,4.190)	3.359(2.077,5.432)
Q4	4.446(3.100,6.377)	4.391(3.013,6.400)	4.758(2.938,7.706)
TyG-WHtR (per 1 unit)	1.685(1.536,1.848)	1.545(1.400,1.705)	1.483(1.313,1.675)
TyG-WHtR (quartile)			
Q1	1.00(Reference)	1.00(Reference)	1.00(Reference)
Q2	1.811(1.214,2.700)	1.502(0.997,2.262)	1.736(1.068,2.822)
Q3	3.200(2.208,4.639)	2.449(1.666,3.599)	2.582(1.618,4.123)
Q4	4.972(3.477,7.110)	3.545(2.445,5.141)	3.400(2.139,5.403)

Model 1: no covariates were adjusted;

Model 2: age, gender, and race were adjusted;

Model 3: age, gender, race, education level, marital status, PIR, diabetes, hypertension, smoking and drinking status were adjusted

**Fig. 2:**
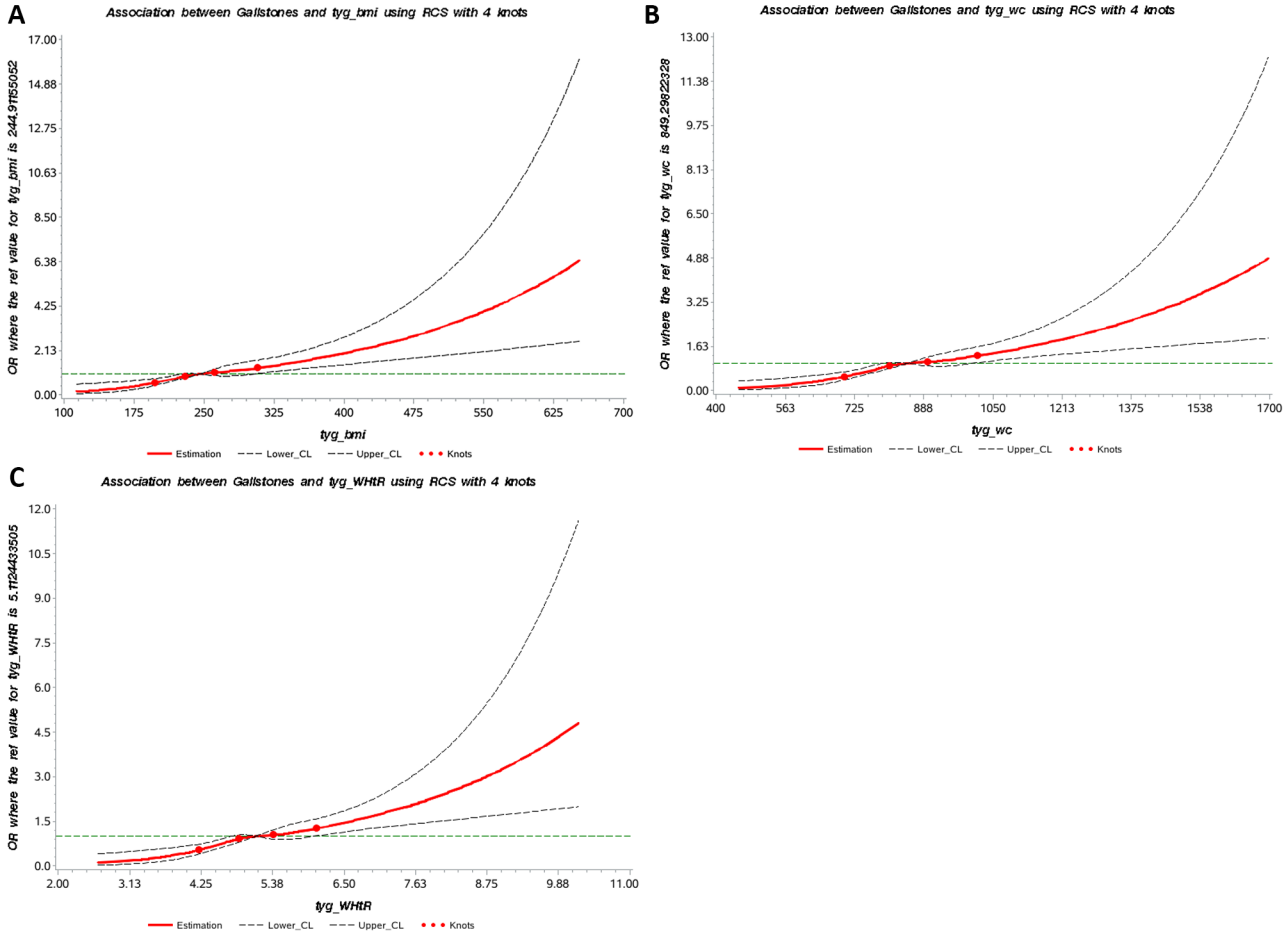
RCS plot of the association between TyG-BMI, TyG-WC, TyG-WHtR and gallstone disease

**Fig. 3:**
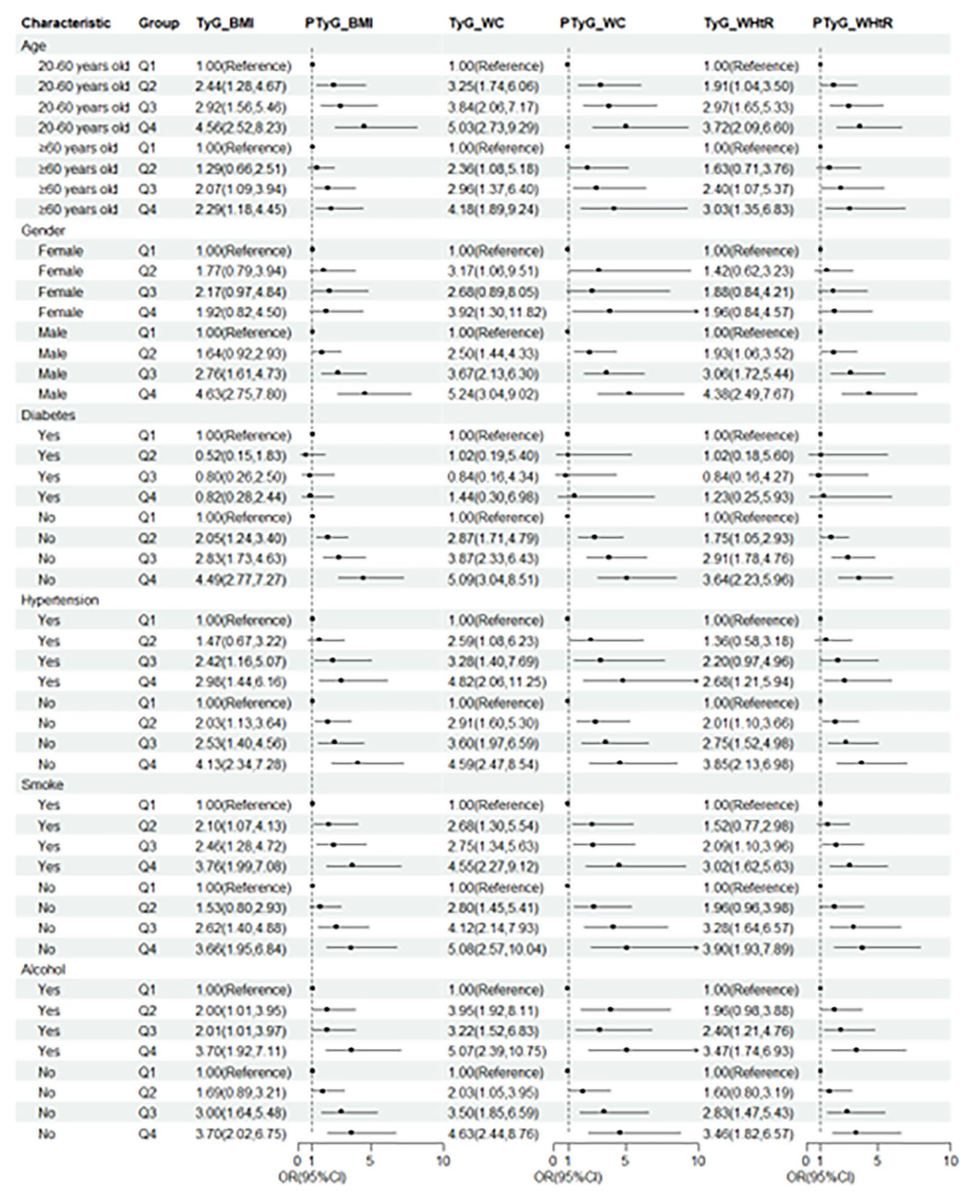
Subgroup analysis of the association between TyG-BMI, TyG-WC, TyG-WHtR and gallstone disease

The RCS plot also presented progressive increases in the risk of gallstone disease as the TyG-related indices rise (Fig. 2). Subgroup analysis revealed that the association between the three TyG-related indices and gallstone disease was more pronounced in people aged 20–60 years, females, with diabetes and hypertension (Fig. 3).

### Comparison of three TyG-related indices

**Fig. 4:**
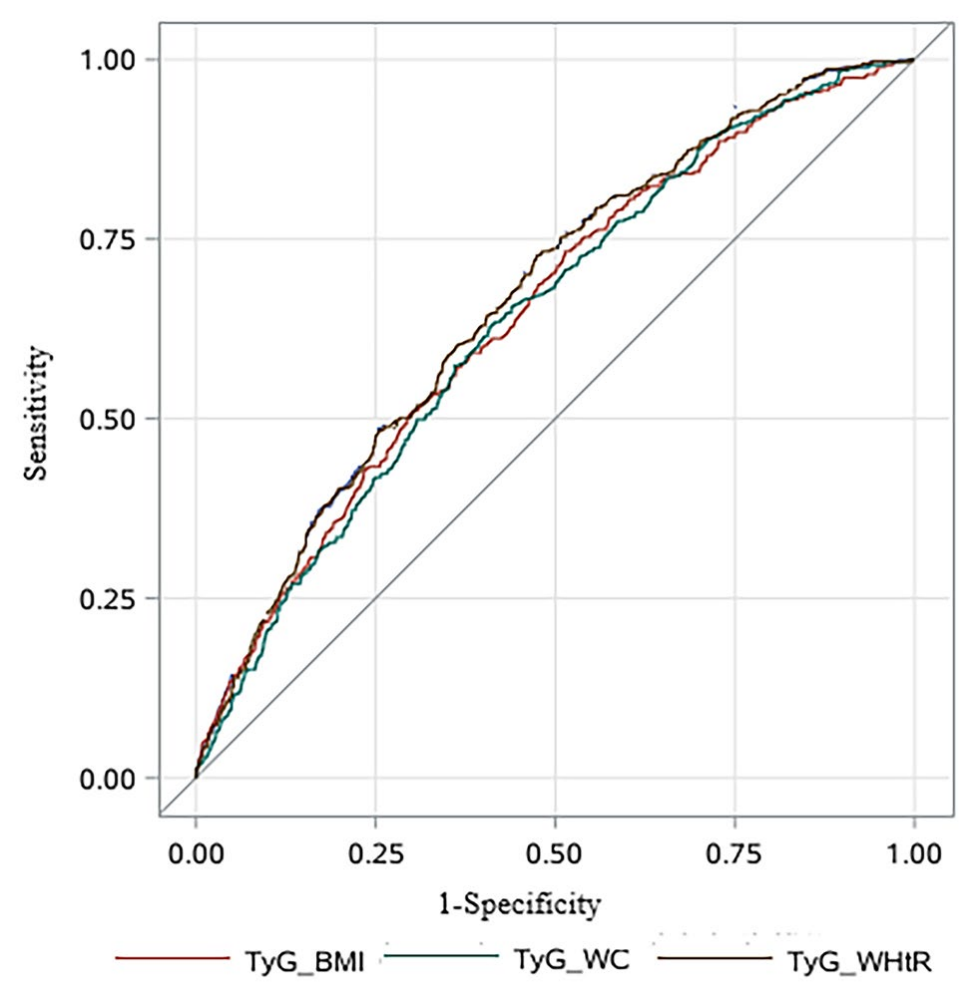
ROC curves of the TyG-related indices for predicting gallstone disease

Table 4 reflected the diagnostic capabilities of TyG-BMI, TyG-WC and TyG-WHtR for gallstone disease. Among these indices, TyG-WHtR demonstrated the best predictive capability, boasting the largest area under the curve (AUC) of 0.667 (95%CI: 0.640, 0.693). TyG-BMI followed with an AUC (95% CI) of 0.647 (0.619, 0.674). Conversely, TyG-WC demonstrated relatively weaker predictive power for gallstone disease, with an AUC (95% CI) of 0.640 (0.613, 0.667). The ROC curves are illustrated in Fig. 4. Utilizing the cutoff point closest to the upper left corner of the ROC curve, the cutoff values for TyG-BMI, TyG-WC, and TyG-WHtR were determined as 260.0, 878.7, and 5.415, respectively. These cutoffs yielded sensitivities of 0.606, 0.630, and 0.602, and specificities of 0.610, 0.590, and 0.637, respectively.

**Table 4:** Performance assessment of the TyG-related indices for the prediction of gallstone disease

Variables	AUC (95%CI)	Cutoff threshold	Sensitivity	Specificity
TyG-BMI	0.647(0.619, 0.674)	260.0	0.606	0.610
TyG-WC	0.640(0.613, 0.667)	878.7	0.630	0.590
TyG-WHtR	0.667(0.640, 0.693)	5.415	0.602	0.637

## Discussion

This extensive cross-sectional population-based study investigated the correlation between TyG-related indices and gallstone disease. Our findings revealed a significant correlation between TyG-related indices (TyG-BMI, TyG-WC, and TyG-WHtR) and gallstone disease, and TyG-WHtR exhibited superior diagnostic capability. These indicess are more accessible and cost-effective, which are important for early identification of gallstone disease and reducing the burden of disease.

While prior researches have not specifically explored the connection between TyG-related indices and gallstone disease, existing literature indicates associations between IR and obesity-related indices with gallstone disease. In a case-control study encompassing 881 subjects, HOMA-IR, a conventional index of IR, demonstrated a correlation with gallstone disease [20]. Additionally, BMI, a measure of general adiposity, was found to double the risk of gallstone disease upon reaching overweight or obesity levels [21–23]. Moreover, a cross-sectional study identified high WC as the most important factor in the risk of gallstone disease [OR (95%CI): 3.84(2.11,7.00)] [24]. Furthermore, a Mendelian randomization study by Zhu et al. similarly indicated an elevated WC was associated with a heightened risk of gallstone disease [25]. Research conducted in Iran and Taiwan found that WHtR, serving as a reliable indicator of central adiposity, emerged as the most significant risk factor for gallstone disease in women [26, 27]. Similarly, TyG-WHtR was found to be the strongest predictor of gallstone disease risk among TyG-related indices in our study. In addition, in a prospective study comprising 88,947 participants, elevated BMI, WC, and WHtR were identified as independent risk factors for gallstone disease [28]. TyG related indices not only integrate anthropometric indicators, but also comprehensively evaluate the metabolic state of the body, thereby enhancing their practicability and accuracy of evaluation.

Prior investigations have delved into elucidating the mechanism underlying the association between IR, obesity, and gallstone disease. Study conducted in a high-risk Hispanic population revealed that IR alters gallbladder function by promoting the production of cholesterol supersaturation bile, thereby leading to gallstone formation [20]. Animal experiments have corroborated these findings, demonstrating that mice with isolated hepatic IR exhibit an increased propensity for cholesterol gallstone formation [29]. The mechanism could be attributed to the heightened expression of biliary cholesterol transporters resulting from the disinhibition of the forkhead transcription factor FoxO1, consequently leading to augmented cholesterol secretion. Another mechanism may be that hepatic insulin resistance reduces the expression of bile acid synthetic enzymes, leading to a lithogenic bile salt profile. The association between obesity and gallstones may be due to increased cholesterol secretion, resulting in cholesterol-supersaturated bile that precipitates as cholesterol gallstones [27]. Impaired gallbladder motility, attributed to reduced sensitivity to cholecystokinin in obese individuals, may contribute to gallstone formation [30]. Furthermore, leptin, a hormone pivotal in obesity development, has been implicated in cholelithiasis formation through its regulation of bile acid metabolism in vitro [31].

Our study holds several key advantages. Firstly, leveraging nationally representative data, we unveiled the correlation between TyG-related indices and gallstone disease, thereby providing novel insights into the potential clinical relevance of these readily accessible indices. Secondly, TyG-related indices are composite measures, providing a comprehensive overview of gallstone-related lipids and metabolism, thereby bolstering the robustness of our findings. Thirdly, through subgroup analyses, we explored the consistency of the relationship between TyG-related indices and gallstone disease across diverse populations. However, there are several limitations to acknowledge in this study. Firstly, the cross-sectional design precludes us from establishing causal relationships between TyG-related indices and gallstone disease. Secondly, despite adjustment for multiple covariates, the potential impact of all confounding factors cannot be entirely mitigated. Thirdly, due to some limitations in the data collection process of the NHANES study, there may be some bias in the determination of gallstones. Future targeted studies are still needed to validate and confirm our findings.

## Conclusions

Our study identified a significant correlation between TyG-related indices (TyG-BMI, TyG-WC, TyG-WHtR) and gallstone disease, utilizing data from a representative cross-sectional study conducted in the United States. Among these indices, TyG-WHtR demonstrated superior predictive capability for gallstone disease. Nevertheless, further validation of these results is warranted through prospective cohort studies.

## Data Availability

Publicly available data sets were analyzed in this study. These data can be found at https://www.cdc.gov/nchs/nhanes/index.htm.
